# Understanding Parahydrogen Hyperpolarized Urine Spectra: The Case of Adenosine Derivatives

**DOI:** 10.3390/molecules27030802

**Published:** 2022-01-26

**Authors:** Kerti Ausmees, Nele Reimets, Indrek Reile

**Affiliations:** National Institute of Chemical Physics and Biophysics, Akadeemia tee 23, 12618 Tallinn, Estonia; kerti.ausmees@kbfi.ee (K.A.); nele.reimets@kbfi.ee (N.R.)

**Keywords:** parahydrogen, NMR, hyperpolarization, metabolomics, signal assignment, adenosines

## Abstract

Parahydrogen hyperpolarization has emerged as a promising tool for sensitivity-enhanced NMR metabolomics. It allows resolution and quantification of NMR signals of certain classes of low-abundance metabolites that would otherwise be undetectable. Applications have been implemented in pharmacokinetics and doping drug detection, demonstrating the versatility of the technique. Yet, in order for the method to be adopted by the analytical community, certain limitations have to be understood and overcome. One such question is NMR signal assignment. At present, the only reliable way to establish the identity of an analyte that gives rise to certain parahydrogen hyperpolarized NMR signals is internal standard addition, which can be laborious. Herein we show that analogously to regular NMR metabolomics, generating libraries of hyperpolarized analyte signals is a viable way to address this limitation. We present hyperpolarized spectral data of adenosines and give an early example of identifying them from a urine sample with the small library. Doing so, we verify the detectability of a class of diagnostically valuable metabolites: adenosine and its derivatives, some of which are cancer biomarkers, and some are central to cellular energy management (e.g., ATP).

## 1. Introduction

Nuclear Magnetic Resonance spectroscopy (NMR) is among the most successful analytical techniques for metabolomics research. NMR has found widespread application in the field due to its high reproducibility, quantitative nature, and the ability to provide relatively simple analyte identification by structural information embedded into NMR signals or comparison with spectral databases [1]. These strengths have allowed NMR metabolomics studies to be conducted in a vast array of research fields, ranging from food science to medical research and biomarker discovery. Yet, all applications are limited by the sensitivity of NMR, excluding the detection of a large and interesting part of the metabolome that appears below the NMR limit of detection (LOD) [2].

Nuclear hyperpolarization techniques have been developed to increase NMR sensitivity by generating non-Boltzmann nuclear polarization, thereby boosting sensitivity [3]. Out of several hyperpolarization techniques available, parahydrogen hyperpolarization has lately garnered attention in the analysis of biological fluids. In particular, the parahydrogen (*p*H_2_) hyperpolarized chemosensing technique [4] has been used for the study of various biological mixtures such as flavor compounds in coffee extracts [4] and whiskey [5]. A modification of the technique has been demonstrated to work in SPE extracts of human urine [6] and in (almost) whole urine [7], allowing for the detection of endogenous urinary metabolites below the LOD of regular NMR.

### 1.1. Parahydrogen Hyperpolarized Chemosensing

**Figure 1:**
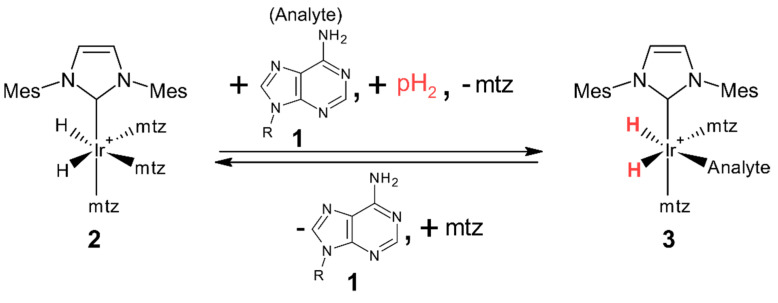
The principle of chemosensing by *p*H_2_-induced hyperpolarization [4]. Complexes **3** are transient in nature, which is necessary for the incorporation of a new analyte and fresh *p*H_2_. Mes denotes 1,3,5-trimethylphenyl group, known as mesityl group; *mtz* denotes 1-methyl-1,2,3-triazol.

Instead of directly measuring NMR signals of analytes **1** (Figure 1), hyperpolarized chemosensing relies on the ability of **1** to reversibly bind to an iridium-based catalyst **2**. Upon binding, complex **3** is formed that presents two *p*H_2_ sourced hydride signals’ doublets (Figure 1) at the −20…−30 ppm region. Chemical shifts of complex **3** hydride resonances are characteristic to each analyte **1** and function as their chemosensors [4]. Intensities of the hydride signals are up to 1000-fold enhanced [6] and the atypical detection region allows for the separation of hyperpolarized dilute analyte signals from the more abundant not-catalyst-interacting analytes. However, it also gives rise to three limitations: analyte scope, quantification, and signal assignment.

Analyte scope of the method is limited by its chemoselectivity, making it less universal than traditional NMR as it detects only analytes capable of binding to **2**. Yet, chemoselectivity can also be viewed as a strength—the method enhances signals which would otherwise be undetectable and enables the resolution of their hydride resonances in the atypical −20…−30 ppm region instead of the crowded 0…10 ppm region [4]. Furthermore, the complexity of published hyperpolarized urine spectra [6,7,8,9] demonstrates that even the catalyst’s limited chemical scope allows for the detection of large numbers of metabolites. The list of known **2** binding analytes is growing and includes, on top of nitrogenous heteroaromatics, pyruvate [10], tagged oligopeptides [11,12], amino acids [13,14], nitriles [15], and sulfur heteroaromatics [16], suggesting the chemoselectivity envelope is wide.

The method is not quantitative in the same way as traditional ^1^H NMR, since multi-pulse NMR detection schemes and analyte-specific complex **3** dissociation kinetics [17], hydrogen exchange rate and relaxation [18] properties are involved. However, similarly to regular 2D NMR [19], quantification can be achieved by analyte-specific calibration curves [4,5,6,7,8,9,13]. When exact values are not necessary, concentrations can be estimated by comparison of signals representing chemically similar analytes [13].

**Figure 2:**
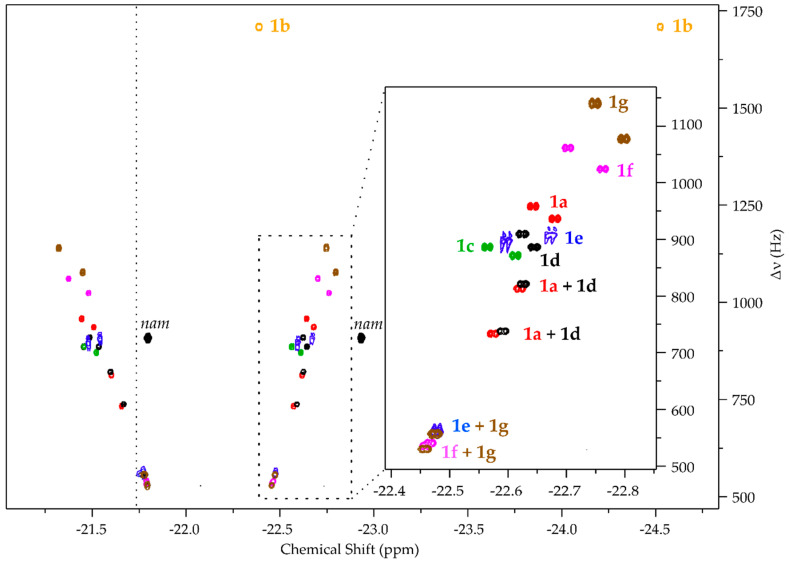
Hyperpolarized 2D ZQ spectra [6] of the adenosines’ library. Spectra of all compounds have been recorded separately, referenced to *nam* signal (using −22.935 ppm for right hand hydride signal [6]) and superimposed. For clarity, all hydride signals for a certain compound are presented in the same color, although the doublet pairs are detected in opposite phase. All analytes (**1a**–**1g**) present at least two clearly resolved signal pairs. The dominating signal of complex **2** (dotted line at −21.74 ppm, see Figure S1 Supplementary Materials) was omitted from the spectra by convolution filtering. See Figure S2 for hyperpolarized hydrides’ 1D spectra of all analytes and Table S1 for chemical shifts of hydride signals.

Signal assignment is arguably the most notable limitation hindering wider adoption of the technique. Since hydride signals do not carry as much structural information as traditional NMR, the only reliable way of assigning signals has proven to be internal standard addition [4,7,9]. The most practical pulse sequence for hyperpolarized chemosensing [6], which can also be carried out as very fast experiments [20], resolves hydride signals (red in Figure 1) according to their mutual zero quantum (ZQ) frequency in the indirect dimension of 2D spectra. Signals of structurally similar analytes have been found to form linear patterns in the 2D ZQ plots ([6] and Figure 2). These patterns aid identification [6,9] but the physical explanation for their formation has not been described yet.

Notably, chemical shifts of hydride signals of different complexes **3** have proven to be largely identical in methanolic urine samples and in simple methanol solutions [7]. Consequently, it should be feasible to compile libraries of analyte signals, which could be applied for signal assignment in complex samples. Herein we present our work on compiling the first such library by establishing a group of valuable metabolites, rationalizing their interactions with a catalyst **2** and applying the small initial library to a biofluid derived sample. We suggest this work presents a practical workflow for understanding complex hyperpolarized spectra and for developing new applications.

### 1.2. Analytical Value of Adenosine Derivatives

Adenine, the nitrogenous base of adenosine, was among the first biological analytes detected by its iridium-bound derivative’s hydride signals [21], suggesting that the study of its different modifications should be feasible. Adenosine has been hyperpolarized by **2** in a SABRE experiment [22], and adenosine detection by hyperpolarized chemosensing has been demonstrated in urine SPE extracts [6] and minimally altered urine [7]. In terms of (bio)analytical utility, the concentrations of adenosine and its methylated derivatives in urine correlate to increased protein turnover in several pathological processes [23], including tumors [24]. The urine of cancer patients is expected to contain elevated concentrations of methylated adenosines [25], such as 1-methyladenosine **1b** (Table 1). N6-methyladenosine **1c** and 2′-O-methyladenosine **1d** have been suggested as cancer biomarkers [26,27] that increase in correlation to tumor size [28]. Urinary concentration of these analytes is in the sub- to low-µM region [29], excluding their detection by regular NMR and rendering them attractive targets for methodology development.

Phosphorylated adenosines (AMP, ADP, and ATP), on the other hand, form the central pinnacle of cellular energy metabolism. While AMP and ADP are also present in urine in low concentrations [29], the more attractive analytical utility of all three may lie in bioenergetics research, uncovering the mechanisms of chemical energy production, and utilization in healthy and malignant cells [30]. ^31^P NMR has traditionally been used for the selective detection of such phosphometabolites in complex cellular extracts, but the lower sensitivity of ^31^P NMR, in combination with the low concentrations of such analytes, has required extensive acquisition times [31]. The detection and resolution of such analytes by hyperpolarized chemosensing would present a further practical application.

## 2. Results

### Building the Library

Our strategy consisted of recording the hyperpolarized hydride spectra of a series of adenosine derivatives to confirm if the chemical modification of an analyte relatively far away from its catalyst binding site would incur detectable differences on hydride chemical shifts. Data acquired for this purpose would also comprise the initial small library that can be used to assign signals in biological samples. According to Wood et al., adenine binds iridium from its 1-, 3- and 9-positions [21] of the purine structure (see structure in Table 1). It can be assumed that adenosine also binds **2** via its nitrogenous base. However, the 9-position is occupied by the ribose moiety in adenosine, leaving only two sites accessible.

The test system used for evaluating a series of adenosine derivatives **1a**–**1g** (Table 1) consisted of typical concentrations of the catalyst and cosubstrate (1.2 mM of **2**, 18-fold excess of *mtz* over iridium) [9], 100 µM of an analyte, and 20 µM of nicotinamide (*nam*). A relatively high analyte concentration was chosen for the convenient detection of the analyte in good SNR conditions and *nam* was used as the internal reference for hydride chemical shifts. Adenosine **1a** has been observed previously to give rise to four pairs of hydride signals [6], which was also seen herein (Figure 2 and Figure S2). Since the catalytic center of **2** is prochiral, it forms two diastereomers upon coordination to a chiral analyte (see discussion in SI of ref. [13]). As **1a** has two possible binding sites, the expected number of hydride signals is doubled, yielding four pairs of signals.

The four pairs of hydride resonances of **1a** complex were two- to three-fold less intense than the signals corresponding to five-fold less concentrated *nam* (Figure S1), since **1a** is distributed among four diastereomeric complexes, whereas all of catalyst bound *nam* (achiral, single binding site) contributes to a single pair of hydride signals. Moreover, the detection conditions (e.g., **2** and *mtz* concentration, sample temperature) may be less optimal for **1a** as hyperpolarization efficiencies are likely to be different due to differences in analyte and hydrogen exchange and relaxation parameters associated with either analyte. Importantly, hydride signal integrals for analytes **1a**–**1d** and **1e**–**1g** were found to deviate two-fold within the two groups of similar analytes (e.g., among **1a**–**1d** and **1e**–**1g**). Between the groups, the largest signal **1a** and weakest **1f** deviated by five-fold. This suggests that approximate quantification of similar adenosines can be carried out by comparing their signals.

In comparison to **1a**, the 2′-O-methylated **1d** gave the most similar signal response, yielding a pattern of four pairs of hydride signals that are slightly shifted (Figure 2). This proves that modification of an analyte relatively far away from the binding site manifests detectable influences on hydride signals. Although half of the hydride signals corresponding to **1a** and **1d** partially overlap, others are well-resolved. N6-methylated **1c**, on the other hand, gives only two signal responses [6], presumably because N6-methylation inhibits N1 binding due to added nearby steric bulk. Confirming the actual binding regimes, however, remains the subject for future work.

N1-methylated **1b** gives the most unexpected spectral pattern, which has also been observed before [6,7]: it seems to give rise to just a single pair of hydride signals that are noticeably shifted in the spectrum. These two signals, upon closer observation, have some internal structure (Figure S3), suggesting that they may be due to two closely overlapping diastereomers. That would explain the number of signals, since one of the catalyst binding sites (N1) is blocked for **1b**, but not their chemical shifts. It is known that N1-methylation of adenine base [32,33] forces it to adopt a different tautomeric (imine) form (Figure S4) and strongly affects its basicity [34]. We suggest this is the reason for the noticeable difference of their catalyst complex hydride chemical shifts, although the exact active catalyst binding site remains unknown.

In the bioenergetics application scenario, detection and resolution of adenosine, AMP, ADP, and ATP is required. It was found that modification of adenosine with different numbers of phosphate groups at its most distant 5′-O-position rendered all four resolvable by their catalyst complex hydride signals. Unlike for **1a**, two of the four diastereomeric complex hydride signal pairs overlap totally for **1e** (AMP; Figure 2 and Figure S5) and partially for **1f** (ADP) and **1g** (ATP; Figure 2 and Figure S5). The same signals also overlap among all three, but the other hydride signal pairs are well-resolved. Unlike for any other analyte, **1e** signals were found to be of irregular shape on the 2D ZQ spectrum. This was caused by a change in hydride signal chemical shifts during 2D acquisition (see comparison of 1D hydride spectra at sample preparation and 1.5 h later in Figure S6). Since *nam* signals remain at the same frequency, we suggest that this change is not caused by processes interfering with the catalyst or *mtz*, but may be the result of gradual deuteration of the analyte during acquisition. A similar process was observed for pyridine by Sellies et al. [13] with similar magnitude changes to the chemical shifts occurring (here 11.5 Hz for the rightmost doublet).

## 3. Discussion

**Figure 3:**
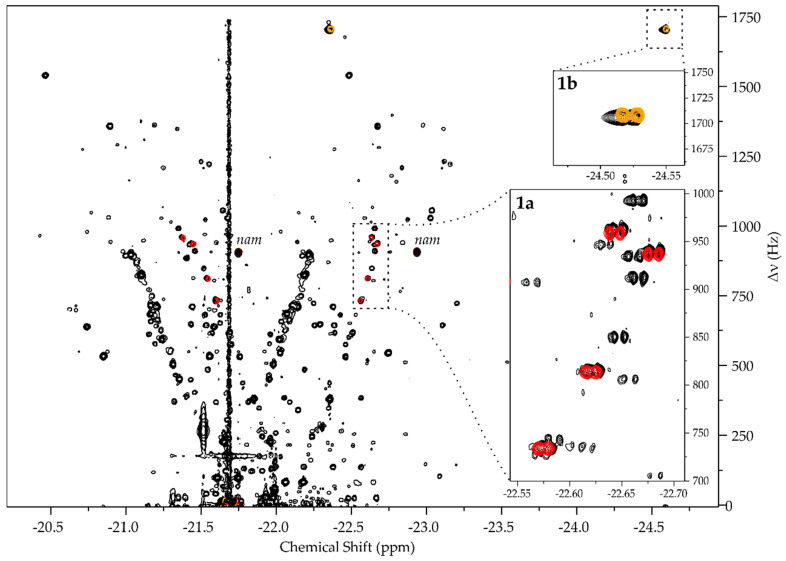
Hyperpolarized 2D ZQ spectrum of urine SPE extract overlayed with spectra of (**1a**) (red) and (**1b**) (beige), referenced to *nam* signal (using −22.935 ppm for right hand hydride signal [6]). All hydride signals of the same analyte are presented in the same color, although the doublet pairs are detected in opposite phase [6].

The acquired information can be used for signal assignment when hydride spectral database is superimposed onto complex spectra of biofluids (Figure 3). That way, signals of compounds in the database could be easily recognized. Herein, this was tested on a urine SPE extract, which was prepared with an SPE protocol modified from previous work [8].

Since the protocol was directed toward the extraction of apolar to mildly polar analytes, the detection of phosphonucleotides **1e**, **1f**, and **1g** was not expected. The retention of other tested analytes was possible, considering **1a**–**1c** were identified by spiking in a similarly prepared SPE extract [6]. Overlaying the database and the experimental spectrum demonstrated an overlap of signals from **1a** and **1b,** whereas at the expected locations of **1c** and **1d**, SNR was too weak to confirm the analytes.

The inability to detect **1c** and **1d** may have been caused by two factors. Firstly, the particular urine sample was obtained from a healthy non-smoking volunteer without any known underlying disease. The lack of medical conditions that are expected to increase urinary methylated adenosine concentrations is likely to contribute to their low abundance in the tested sample. There are alternative SPE procedures that would provide more effective retention of adenosines by covalent bonding to the ribose *cis*-diol moiety [35], if detection of the less abundant derivatives is desired.

Secondly, since the work was aimed toward verifying the resolution of the analytes and confirming the applicability of the concept of libraries, maximum sensitivity was not a priority. Consequently, all spectra were acquired with 50% *p*H_2_ and without a cryoprobe, which has been commonly applied in prior instances of hyperpolarized chemosensing in urine samples [6,7,8,9,13]. A cryoprobe would further increase sensitivity by three- to four-fold [36], while increasing *p*H_2_ enrichment to 100% would result in an additional threefold increase [37]. Hence, an order of magnitude sensitivity increase would be available by upgrading both the *p*H_2_ source and the NMR probe.

All things considered, the ability to assign low-µM **1a** and **1b** on a room temperature probe by library overlap is a positive result that demonstrates the feasibility of the strategy. Moreover, measured chemical shifts of **1a**, **1b**, and **1c** hydride signals coincide with an earlier report [6], suggesting that such libraries would have universality over different instrumentation and different SPE sample preparation procedures. Hereby we have demonstrated the following concepts:

- *p*H_2_ hyperpolarized chemosensing is remarkably sensitive to relatively small changes in analyte structure, allowing for the resolution of series of structurally highly similar metabolites, including closely related isomers (i.e., **1b**, **1c**, **1d**).
- Libraries (Table S1) can be built for the assignment of specific families of metabolites. With a few exceptions (e.g., **1b**), the observation of Sellies et al. [6] that signals of similar analytes form linear patterns in the ZQ spectra holds.
- Libraries can be applied by a straightforward superposition of experimental and database spectra.
- The databases would have universality across different instruments, laboratories, and variations in sample preparation procedures.

This work adds to the *p*H_2_ hyperpolarization toolbox, presenting a strategy for understanding the complex hydride spectra from the parahydrogen hyperpolarized chemosensing experiment. Building practical applications by using the spectral data provided herein, and adding to this data, will be the focus of our future work.

## 4. Materials and Methods

NMR experiments were conducted at sample temperature of 25 °C on an 800 MHz Bruker Avance III spectrometer equipped with a 5 mm TXI probe. Hyperpolarization was carried out in 5 mm Norell S-5-500-IPV-7 pressure tubes with a previously described hyperpolarization setup [9]. *p*H_2_ was prepared in flow, as described previously [9]. Hyperpolarized 1D SEPP spectra (Figures S1–S3, S5 and S6) and hyperpolarized 2D ZQ spectra (Figure 2 and Figure 3) were acquired with previously published pulse sequences [6], utilizing high power hard pulses and shaped *reburp* pulses that covered all signals of interest. One-dimensional spectra were acquired in 64 scans. Two-dimensional data were acquired in 320 increments over 2500 Hz in f_1_, except for **1b** and urine SPE extract, for which 512 increments were acquired over 3500 Hz spectral width. Two scans per increment were acquired in all cases. Processing was done following earlier described principles [6,9] with Mestrenova 14.2 software.

Waters Oasis HLB^®^ 6 mL / 200 mg cartridges were used for solid phase extraction (SPE). Methanol used to activate the cartridges was obtained from Honeywell Riedel-de Haën™. Analytes were eluted with, and experiments were conducted in, methanol-d_4_ obtained from Deutero Gmbh. 1-methyladenosine was acquired from Cayman Chemicals. Nicotinamide and adenosine were acquired from TCI Chemicals. N6-methyladenosine and 2′-O-methyladenosine were acquired from Carbosynth. AMP, ADP, and ATP were obtained from Sigma Aldrich. All chemicals were used as supplied. Catalyst **2** precursor [Ir(Cl)(COD)(Imes)] and cosubstrate 1-methyl-1,2,3-triazol (*mtz*) were synthesized in-house by the same methods [6,7,9] as previously described.

1…2 mM stock solutions of all analytes in methanol-d_4_ were prepared gravimetrically. NMR samples were prepared by adding 150 µL of 4.8 mM solution of [Ir(Cl)(COD)(Imes)], 13 µL of 1 M stock solution of *mtz*, and 37 µL of methanol-d_4_ to a pressure tube, pressurizing the sample under 5 bar of H_2_, shaking the tube and allowing the catalyst to convert to its active form **2** in 2 h. Then, appropriate amounts of analyte stock solutions and methanol-d_4_ were added to reach 1.2 mM concentration of **2**, 20 µM of *nam* and 100 µM of the particular analyte in 600 µL of total sample volume. The tube was connected to the hyperpolarization setup [9] for NMR experiments.

A urine sample was collected from a healthy non-smoking volunteer as morning first midstream urine and frozen at −80 °C. Prior to analysis, the sample was thawed over a room temperature water bath, pH-adjusted to 8.0 with 1 M NaOH and centrifuged for 12 min at 1825× *g*. SPE of the resulting urine sample was carried out by activating the SPE cartridge with 5 mL of methanol, conditioning it with 3 mL of 10 mM pH 8.0 phosphate buffer., loading 5 mL of urine and eluting it by light nitrogen overpressure above the cartridge (approx. 2–3 mL/min). The cartridge was washed with 3 mL of 10 mM pH 8.0 phosphate buffer and dried for 30 min with a nitrogen flow generated by 1 bar N_2_ overpressure above the cartridge. Finally, analytes were eluted by adding 1.2 mL of methanol-d_4_, which yielded approximately 800 µL of extract. For hyperpolarization experiments, 450 µL of the extract was mixed with the preactivated solution of **2** and *mtz* (1.2 mM final concentration of **2**, 18-fold excess of *mtz*).

## Figures and Tables

**Table 1:**
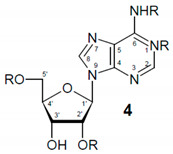
Adenosines used in the study.

Analyte	No	R @ 1	R @ 6	R @ 2′	R @ 5′
Adenosine	**1a**	H	H	H	H
1-Methyladenosine *	**1b**	CH_3_	H	H	H
6N-Methyladenosine	**1c**	H	CH_3_	H	H
2′-O-Methyladenosine	**1d**	H	H	CH_3_	H
AMP	**1e**	H	H	H	PO_3_H
ADP	**1f**	H	H	H	PO_3_-PO_3_H
ATP	**1g**	H	H	H	PO_3_-PO_3_-PO_3_H

* **1b** likely adopts the neutral imino conformer structure in methanol (see Figure S4).

## Data Availability

The data presented in this study are openly available in Mendeley Data at 10.17632/3cwxd4d472.1.

## Supplementary Materials

The following supporting information is available online, Figure S1: Hyperpolarized 1D hydride spectrum of adenosine **1a** sample; Figure S2: Hyperpolarized 1D spectra of the hydride spectral regions of the same samples as main text Figure 2; Figure S3: The structure of **1b** hydride signals; Figure S4: 1-Methyladenosine **1b** neutral iminium tautomer; Figure S5: Comparison of 1D hydride spectra of AMP (**1e**), ADP (**1f**) and ATP (**1g**); Figure S6: 1D hyperpolarized hydride spectra of the same **1e** containing sample within minutes from preparing the sample (red) and 1 h later (black); Table S1: Library of hydride chemical shifts of complexes **3** for adenosine derivatives **1**.

## Sample Availability

Compounds **1a**–**1g** and *nam* were acquired commercially. Samples of catalyst **2** precursor [Ir(Cl)(COD)(Imes)] and cosubstrate 1-methyl-1,2,3-triazol (*mtz*) are available from the authors.
