# Supplementary material for: Understanding Parahydrogen Hyperpolarized Urine Spectra: The Case of Adenosine Derivatives
Source: Molecules. 2022 Jan 26;27(3):802. doi: 10.3390/molecules27030802 (PMC8837968; doi:10.3390/molecules27030802)
Supplement: Supplementary file 1 [file molecules-27-00802-s001.zip › molecules-1560518-supplementary.pdf]

Supporting information

Understanding parahydrogen hyperpolarized urine spectra: the case of adenosine derivatives

Kerti Ausmees, Nele Reimets, Indrek Reile*

National Institute of Chemical Physics and Biophysics, Akadeemia tee 23, 12618 Tallinn, Estonia

Supplementary figures

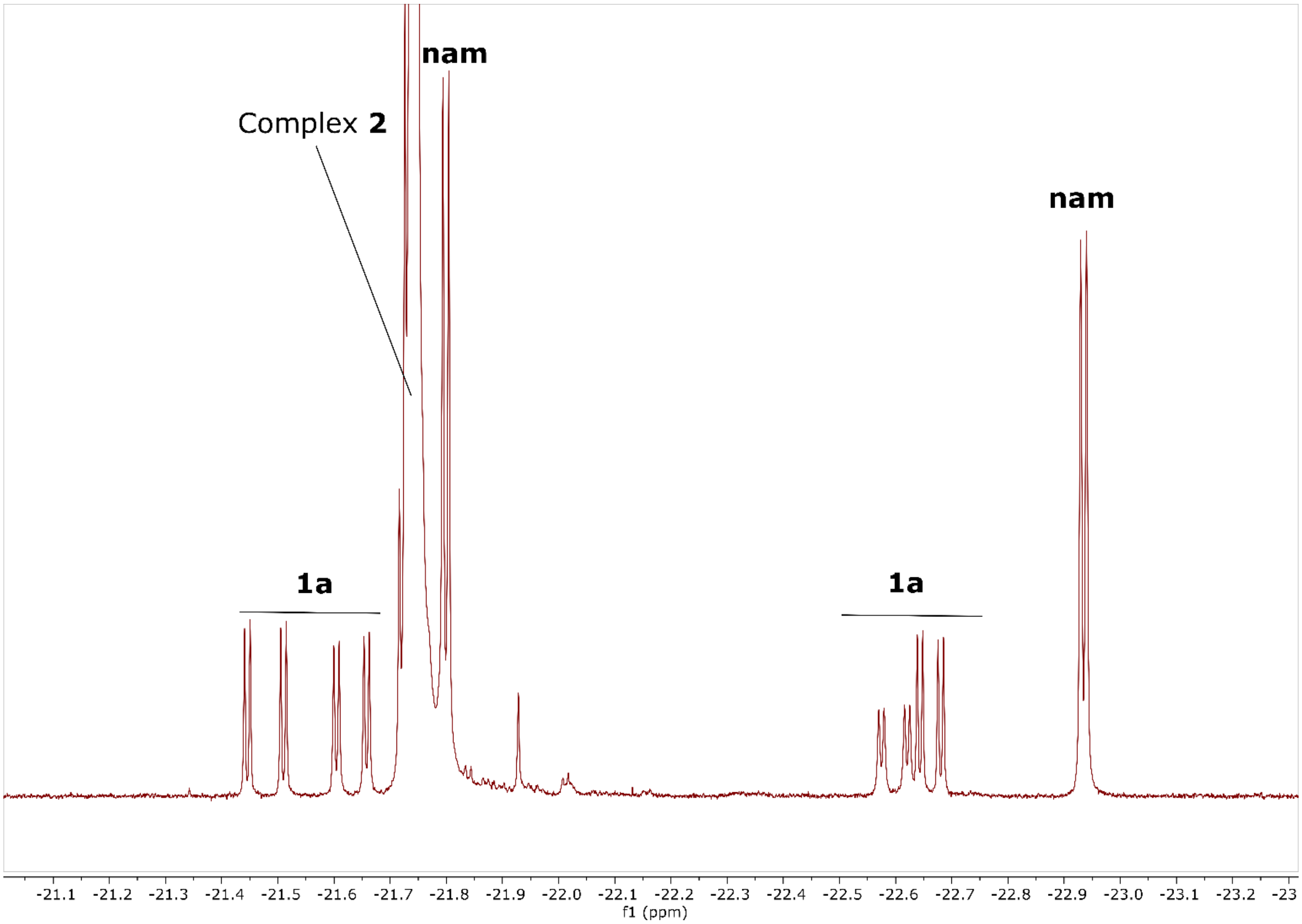

Figure S1. Hyperpolarized 1D hydride spectrum of adenosine **1a** sample, recorded with the same SEPP pulse sequence as in earlier publications [1,2].

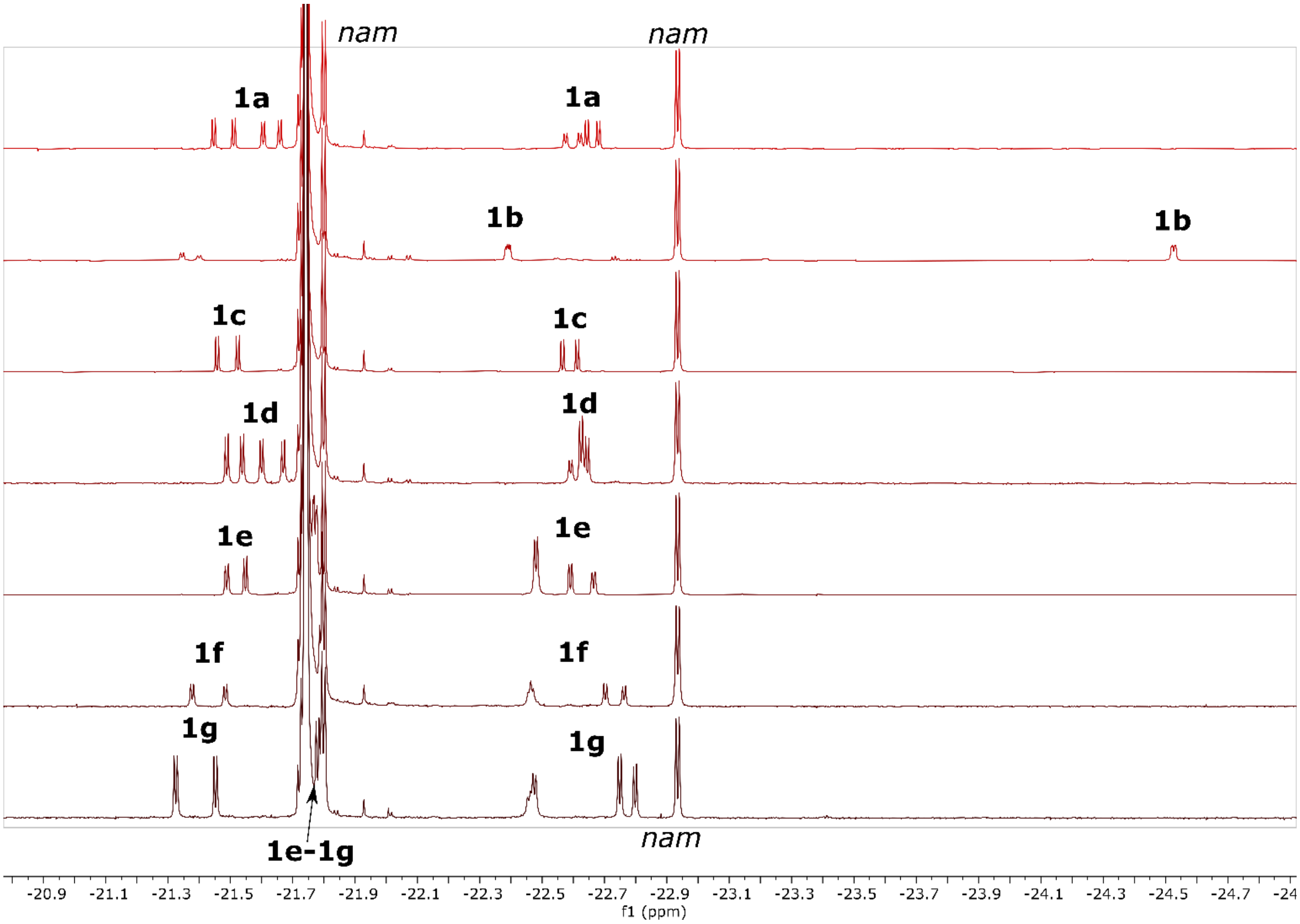

Figure S2. Hyperpolarized 1D spectra of the hydride spectral regions of the same samples as main text Figure 2. All spectra are referenced according to *nam* signal at -22.935 ppm.

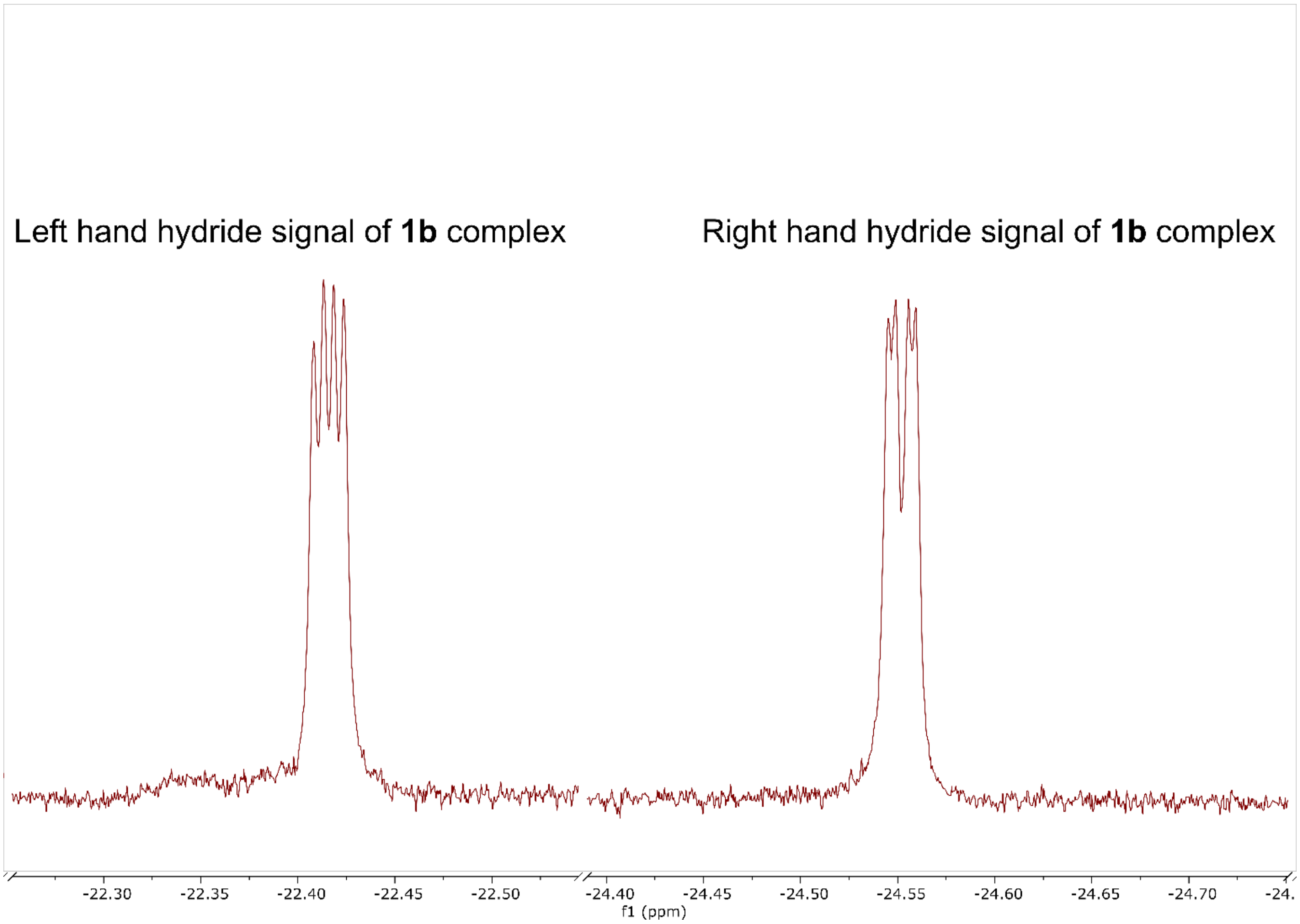

Figure S3. The structure of **1b** hydride signals. Both can be explained by two closely resonating doublets.

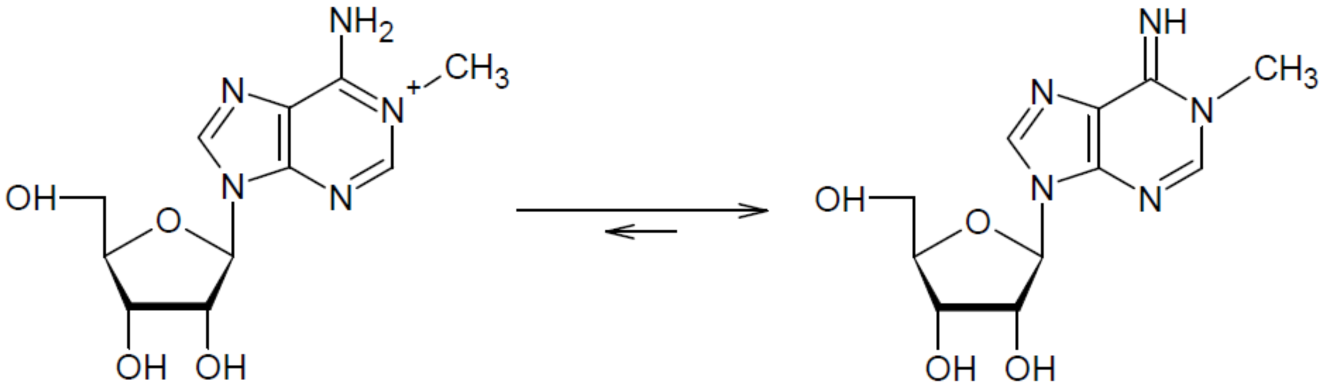

Figure S4. 1-Methyladenosine **1b** is likely to adopt the structure of a neutral iminium tautomer (right) in the methanolic sample environment. This may explain the large difference in hydride chemical shifts, compared to all other tested adenosine derivatives. Moreover, since the metal center on **2** is also positively charged, the lefthand structure would inhibit the analyte from accessing the catalyst due to electrostatic repulsion.

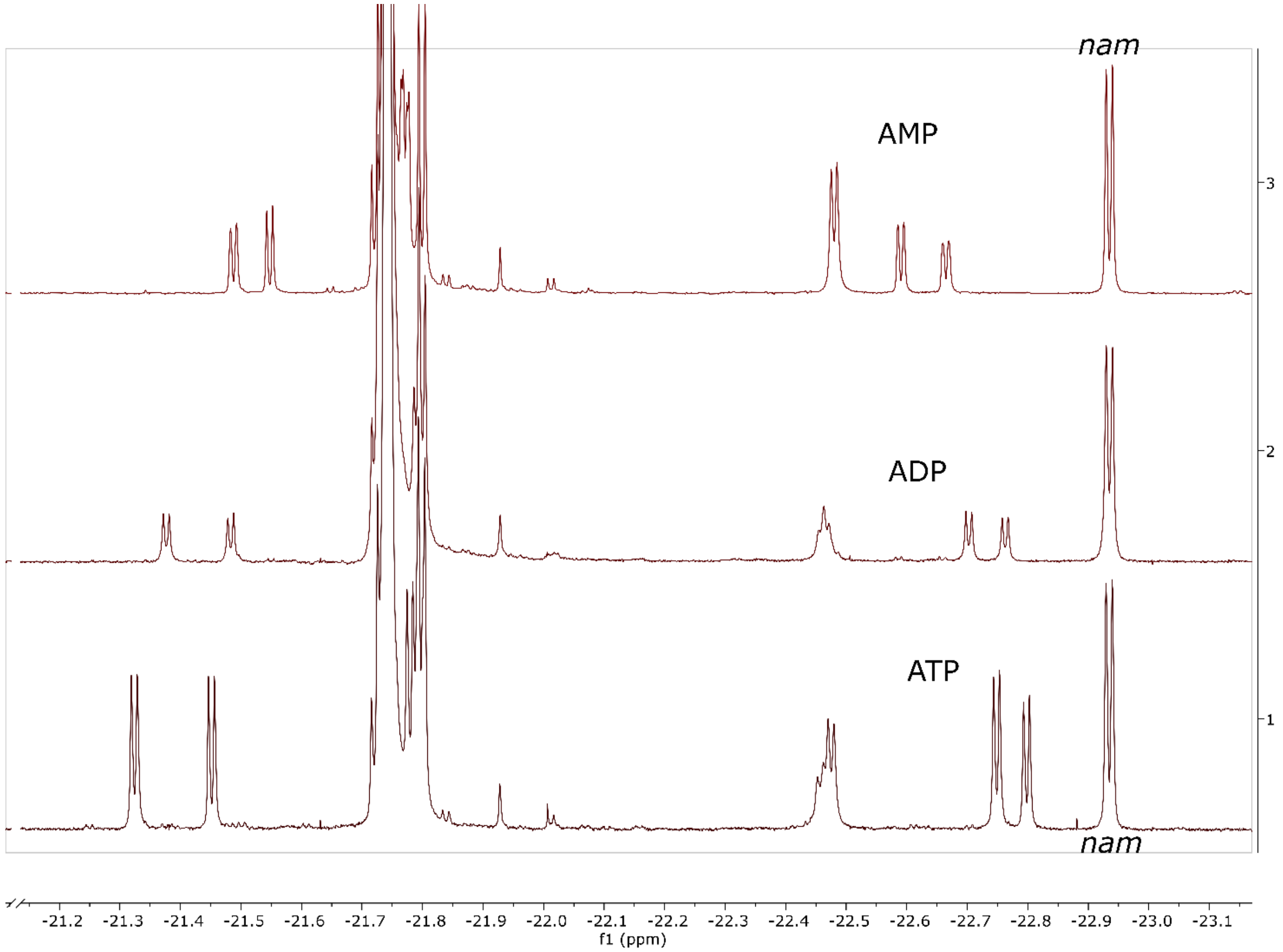

Figure S5. Comparison of 1D hydride spectra of AMP (**1e**), ADP (**1f**) and ATP (**1g**). All can be resolved based on signals between -22.55…-22.85 ppm (see main text Figure 2), or by the left-hand coupling partners of these signals.

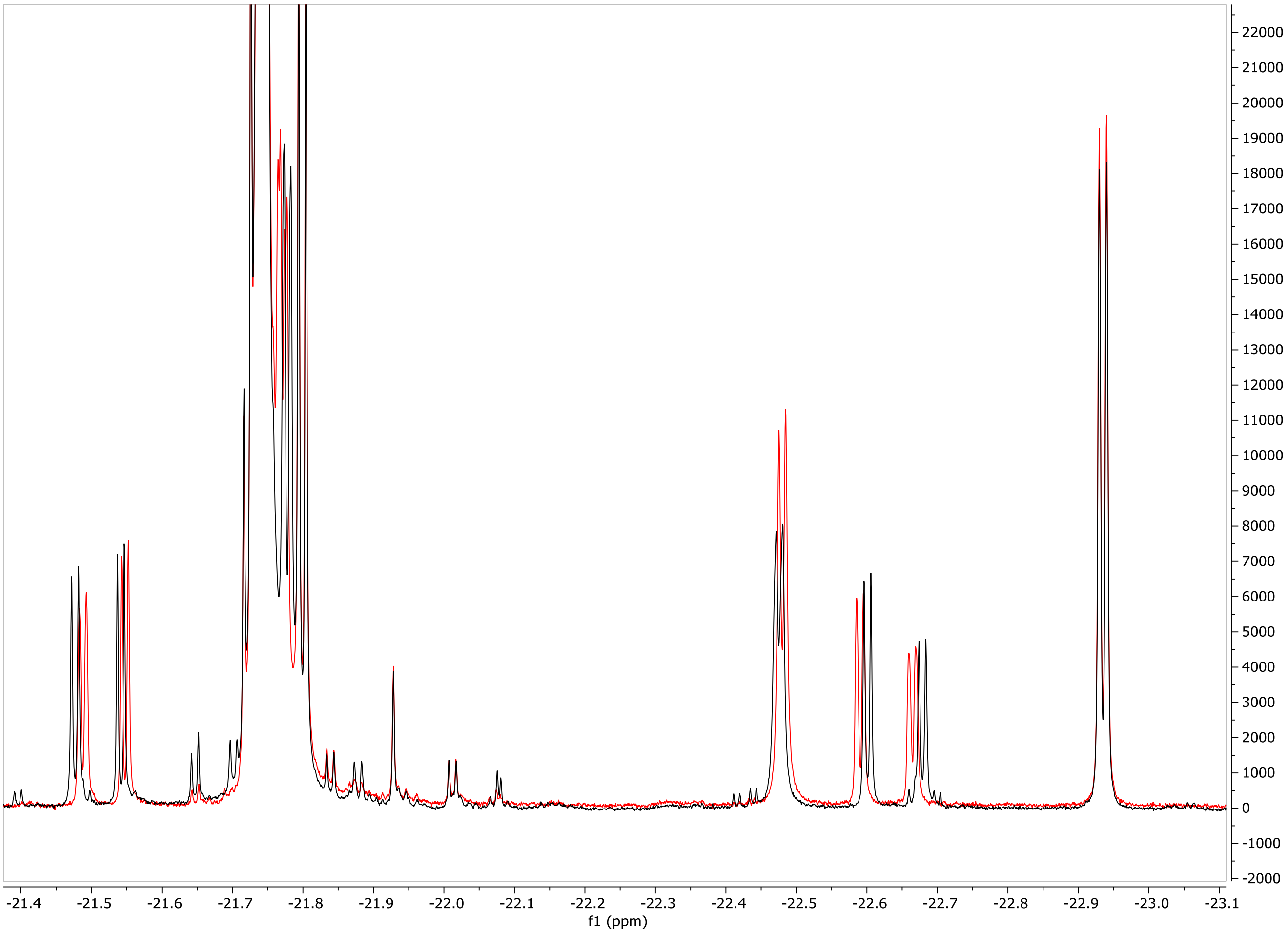

Figure S6. 1D hyperpolarized hydride spectra of the same **1e** containing sample within minutes from preparing the sample (red) and 1 h later (black), during which time the sample was bubbled with *p*H_2 and a 2D ZQ spectrum (main text Figure 2) was recorded. Notice how nicotinamide internal standard signal (-22.935 ppm) and impurity signals at around -22 ppm remain unperturbed.

Table S1. Library of hydride chemical shifts of complexes **3** for adenosine derivatives **1**.

Analyte	Left hydrides (ppm)	Right hydrides (ppm)	J (Hz)
**1a**	-21.510	-22.680	7.8
	-21.446	-22.644	7.6
	-21.605	-22.621	7.3
	-21.658	-22.575	7.6
**1b**	-22.393	-24.528	8.3
	-22.387	-24.524	8.6
**1c**	-21.523	-22.613	7.8
	-21.457	-22.565	7.6
**1d**	-21.534	-22.645	7.6
	-21.488	-22.624	7.8
	-21.600	-22.624	7.6
	-21.669	-22.591	7.6
**1e**	-21.547	-22.663	7.8
	21.488	-22.590	7.8
	-21.770	-22.480	7.3
**1f**	-21.483	-22.763	7.6
	-21.377	-22.703	7.6
	-21.795	-22.468	NA
**1g**	-21.451	-22.798	7.8
	-21.324	-22.748	7.8
	-21.779	-22.475	7.6
	-21.798	-22.457	7.6

References

1. Sellies, L.; Reile, I.; Aspers, R.L.E.G.; Feiters, M.C.; Rutjes, F.P.J.T.; Tessari, M. Parahydrogen induced hyperpolarization provides a tool for NMR metabolomics at nanomolar concentrations. *Chem. Commun.* **2019**, *55*, 7235–7238, doi:10.1039/C9CC02186H.

2. Reimets, N.; Ausmees, K.; Vija, S.; Reile, I. Developing Analytical Applications for Parahydrogen Hyperpolarization: Urinary Elimination Pharmacokinetics of Nicotine. *Anal. Chem.* **2021**, *93*, 9480–9485, doi:10.1021/acs.analchem.1c01281.
